# No Evidence That Abstract Structure Learning Disrupts Novel-Event Learning in 8- to 11-Month-Olds

**DOI:** 10.3389/fpsyg.2019.00498

**Published:** 2019-03-08

**Authors:** Rachel Wu, Ting Qian, Richard N. Aslin

**Affiliations:** ^1^Department of Psychology, University of California, Riverside, Riverside, CA, United States; ^2^Department of Psychology, Princeton University, Princeton, NJ, United States; ^3^Haskins Laboratories, New Haven, CT, United States

**Keywords:** infancy, eye-tracking, learning, object learning, sequence learning

## Abstract

Although infants acquire specific information (e.g., motion of a specific toy) and abstract information (e.g., likelihood of events repeating), it is unclear whether extraction of abstract information interferes with specific learning. In the present study, 8- to 11-month-old infants were shown four audio-visual movies, either with a mixed or uniform presentation structure. Learning of abstract information was operationally defined as the looking time to changes in presentation structure of the movies (mixed vs. uniform blocks), and learning of specific information was defined as the looking time to changes in content in the four movies (object properties and identities). We found evidence of both specific and abstract learning, but did not find evidence that extraction of the presentation structure (i.e., abstract learning) impacts specific learning of the events. We discuss the implications of the costs and benefits of the interaction between abstract and specific learning for infants.

## Introduction

In the natural environment, informative regularities occur at both specific and abstract levels. For example, a sister can teach an infant about how to build a specific snowman with Legos multiple times in a haphazard way (different sequence each time), whereas a brother can teach an infant about how to play with each of his puppets in a very structured way, where he finishes talking about one puppet before moving on to the next puppet. In this example, infants can learn about the *specific* information related to Legos and puppets (e.g., which puppet likes hot dogs and how to construct a Lego snowman), as well as general (*abstract*) characteristics about how the information about Legos and puppets is presented (i.e., pattern of the presentation: haphazard vs. structured). Intuitively, one might predict that learning in a structured way, rather than haphazard way, may enhance specific learning in infants, and that changing structures may disrupt learning. Indeed, many infant studies on learning present repeated information in blocks. Forming accurate representations of the environment utilizes both specific learning and abstract learning. Specific learning is based on the content of the events that the infant experiences (e.g., motion trajectories, spatial localization, audio-visual binding), whereas abstract learning relates to principles inferred from the observed input and is characterized by generalizing from the specific instances (e.g., repetition, categorization, rule learning, context learning).

Decades of research using looking time measures have demonstrated infant competencies at both of these levels of learning (Mareschal and Quinn, 2001; Aslin, 2007; Aslin and Newport, 2012). Classic habituation studies present infants from birth with a single series of identical familiarization trials followed by one or two test trials (e.g., switch trials that present the same stimuli as during familiarization with a change or swap in one feature). These studies with a single “block” of habituation trials measure discrimination of events, and therefore, *specific learning* of content within the repeating events in a block (e.g., differentiating between an object making a familiarized sound vs. the same object making a new sound). These same looking time techniques also can be used to measure abstract learning by displaying variable stimuli prior to the post-familiarization test phase (e.g., Fagan, 1972; Marcus et al., 1999). For example, Marcus et al. (1999; see also Gomez and Gerken, 1999) presented 7-month-old infants with phoneme patterns, such as le-di-di and wo-fe-fe, and although the specific phonemes changed, the overarching abstract structure (e.g., ABB) remained the same. Infants discriminated a change in the abstract structure (e.g., ABB vs. ABA) even when the test stimuli were unfamiliar (e.g., ba-po-po vs. ba-po-ba), thereby showing infants’ ability to learn rule-like higher-level patterns, such as whether objects or features tend to repeat or change.

Although specific and abstract learning have been documented separately in infancy based on numerous looking time studies (for recent examples, see Basirat et al., 2014; Werchan et al., 2015; Ferguson et al., 2018), infants also use abstract knowledge to constrain specific learning in useful ways. For example, 3-month-old infants use the repetition of contextual cues to make inferences about a specific instance (e.g., an interesting event), such as knowing when to trigger an interesting event (Rovee-Collier et al., 1985; see also Tummeltshammer and Amso, 2018). Rovee-Collier et al. (1985) found that the presence of a crib bumper that was maintained from training to test helped 3-month-olds remember the association between kicking and making a mobile move. These findings are aligned with those of young children’s use of object and contextual features to determine an object’s label (1.5- to 4-year-olds; Landau et al., 1988; Samuelson and Smith, 1998; Vlach and Sandhofer, 2011; Wu et al., 2011). Nine-month-olds use specific instances in speech to determine appropriate abstractions (e.g., language rules; Gerken, 2006, 2010), but if there are multiple possible abstractions given a set of stimuli, abstract learning may be attenuated (e.g., Gerken and Quam, 2016).

To our knowledge, no study yet has asked whether changing a recently learned abstract rule disrupts learning of specific events that follow that new rule. It is still unclear whether exposure to a changed rule in novel situations leads to costs in learning new specific information. This issue is important to investigate in infants because if it were the case, then abstract information that is incongruous to existing abstract knowledge could be a barrier to learning specific instances in infancy, as it can in adulthood, such as when the stimulus presentation in the lab setting violates norms in the natural environment (e.g., Kemp and Tenenbaum, 2009; Schwabe and Wolf, 2009; Green et al., 2010; Nako et al., 2014; Orhan and Jacobs, 2014). Returning to the earlier example, if the infant’s sister started teaching the infant about her Mr. Potato Head in a structured rather than a haphazard way (e.g., maintaining a sequence of parts that go in the Mr. Potato Head), would the change at an abstract level disrupt the infant’s ability to learn about the specific features of Mr. Potato Head? On the one hand, it may be possible that learning about specific content under a similar structure may facilitate future specific learning (Marcus et al., 2007), and changing the abstract structure may disrupt specific learning, in line with the idea that maintaining contexts can facilitate learning (e.g., Rovee-Collier et al., 1985; Vlach and Sandhofer, 2011; see also Tenenbaum et al., 2011). On the other hand, specific learning may be unaffected by abstract learning if they operate in parallel.

### The Present Study

**FIGURE 1:**
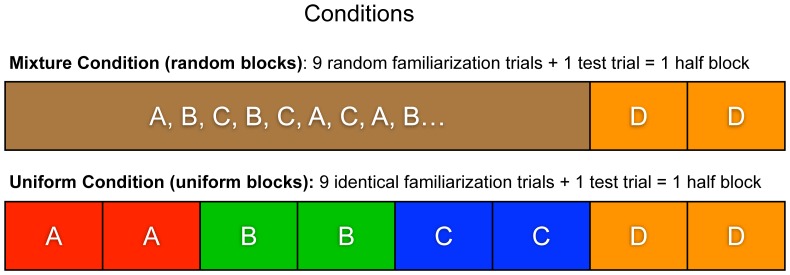
Trial sequence of Mixture (random blocks) and Uniform (uniform blocks) conditions. An example of a sequence of ABC trials in the Mixture condition is presented in the figure. For all of the infants in the Mixture condition, these trials were presented in a random order (e.g., A, B, C, C, B, A or A, C, B, B, A, C, etc). Half blocks consisted of nine familiarization trials and one test trial. In the Uniform Condition (bottom panel), four blocks (eight half blocks) are depicted, where each block presented only A, B, C, or D events. In the Mixture Condition (top panel), four blocks are also depicted, where each of the first three blocks showed A, B, and C events, followed by a D-only block.

The present study used multiple exposure phases within one laboratory session to investigate how generalization to higher-level trial structure (*abstract learning*, i.e., learning about the presentation structure of four movies) affects the learning of trial content (*specific learning*, learning about the content of the four movies) during infancy. There were four types of events (i.e., movies) across the session, with infants in the Mixture condition seeing a randomized ordering of three of the events (i.e., A, B, C) in each of the first three blocks, followed by a uniform fourth block consisting of only the fourth event (i.e., D) (Figure 1). Infants in the Uniform condition were presented with the same four events across four sequential blocks, and each of the four blocks contained only a single event (Figure 1). Thus, in the Mixture condition there were three blocks containing a diversity of events followed by a test block containing only one novel event. In the Uniform condition, all four blocks were uniform. Note that in both conditions the final test block was identical – it consisted of a single novel event repeated many times. Overall, there were four blocks consisting of 20 trials per block (18 familiarization and 2 test trials), and the four events were shown across the four blocks.

Abstract learning was operationalized as discrimination of (i.e., dishabituation to) the novel presentation structure in the Mixture condition because the presentation structure shifted from a randomized ordering of three events (A, B, C) to a repeated single event (D) in the test block. By contrast, abstract learning in the Uniform condition was operationalized as generalization (i.e., the absence of dishabituation) in the test block (D) after habituation to three uniform blocks (A, B, C). Thus, in the Mixture condition a failure of abstract learning would lead to the absence of dishabituation in the test block, and in the Uniform condition to the presence of dishabituation in the test block.

Specific learning was operationalized as dishabituation to a feature change in one of the four events (A, B, C, D) during “switch” trials, which were presented after every 9 trials in each block, which contained 18 familiarization trials and 2 test trials. The four feature changes presented across four audio-visual events were object color, motion, audio-visual binding, and physical properties (i.e., squishable). Our critical measure for assessing the influence of abstract learning on specific learning was the looking time to the switch test trials (trials 10 and 20) during the final test block where a novel event (D) was presented. Again, this final test block was identical in the two conditions.

Based on numerous prior studies on specific and abstract learning, we predicted that infants in our study would exhibit both specific and abstract learning. For specific learning of all four audio-visual events, we predicted that infants in both the Mixture and Uniform conditions would discriminate the novel switch trial from the familiar trials that immediately preceded it. For abstract learning, we predicted that infants in the Uniform condition would treat the presentation structure of Event D as expected, but that infants in the Mixture condition would find the presentation structure of Event D to be unexpected and dishabituate. We also predicted an interaction between abstract learning and specific learning in the Mixture condition, where a change in how the blocks are structured (from mixture to uniform) would decrease specific learning due to a mismatch in the expected and actual presentation formats. In other words, we anticipated that noticing a change between the presentation structure of the first three blocks (Events A, B, and C) and the final block (Event D) in the Mixture condition would decrease looking times during the switch trials in relation to the familiarization trials in the final block. If abstract learning does not disrupt specific learning in infants for novel stimuli, then infants would still dishabituate to the switch test trial for Event D, and the looking times to the test trials in Event D would not differ between the Mixture and Uniform conditions. Critically, the presentation of the final test block was identical across both conditions (i.e., Event D was presented in uniform blocks). Therefore, any looking time differences between conditions in this final test block would be due to differences in the presentation structure over the first three blocks.

## Materials and Methods

### Participants

Thirty-two infants (16 infants per condition) participated in this experiment in either the Mixture condition (randomly presented events in the first three blocks; *M* = 9 months and 4.1 days, range: 7 months and 20 days to 11 months and 10 days, 7 males) or the Uniform condition (events grouped into blocks of A–D; *M* = 9 months and 8.6 days, range: 7 months and 15 days to 11 months and 13 days, 5 males). Of the infants whose race and ethnicity were reported, 97% of the infants were at least half Caucasian, and 3% were half Black, 13% half Asian, 3% half Hawaiian/Pacific Islander, 3% half Native American, and 6% Hispanic. The pre-determined stopping point for recruitment was based on prior research using similar sample sizes ranging from 11 to 20 infants (e.g., Baillargeon, 1987; Richardson and Kirkham, 2004; Rakison, 2005; Xu and Garcia, 2008). A relatively wide age range was included in the study to accommodate the variety of abilities related to specific and abstract learning documented in prior studies, especially in relation to the four events that we included. Three additional infants were tested but were excluded from the final sample because of fussiness (*N* = 2, one in each condition did not complete the entire experiment) or equipment error (*N* = 1 in the Mixture condition). This low drop-out rate compared to typical infant looking-time studies may have been due to the interesting nature, timing, and variability of the stimuli (e.g., Stets et al., 2013), as well as the right level of predictability vs. unpredictability (e.g., Kidd et al., 2012). Infants were recruited via hospital birth records and were given an infant-sized t-shirt and $10 for their participation. All parents of infants provided written informed consent, and the study conformed to the United States Federal Policy for the Protection of Human Subjects.

### Stimuli

**FIGURE 2:**
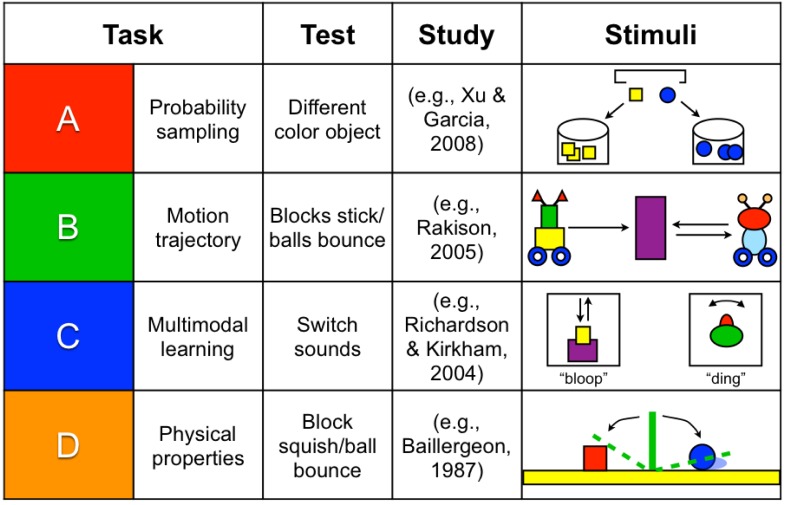
Sample stimuli A–D. All events were counterbalanced for all infants (i.e., Event A for one infant was Event D for another infant).

This study tracked infants’ looking time to four different types of events (i.e., movies) commonly used in infant studies: *probability sampling* (e.g., Xu and Garcia, 2008), *motion trajectory* (e.g., Rakison, 2005), *multimodal binding* (e.g., Richardson and Kirkham, 2004), and *object solidity* (e.g., Baillargeon, 1987; Figure 2). The probability sampling task displayed yellow blocks and blue balls falling from the top of the screen into corresponding buckets on the left and right side. A dropping sound was played as the objects simultaneously fell into the buckets. The dropping sound and action lasted 10 s. In the motion trajectory task, infants were shown two simultaneous wheeled toys (round or rectangular) that rolled from the left or right to the center of the screen with an accompanying rolling sound. One toy stuck to the median (at 5 s into the movie), while the other toy simultaneously bounced off the median and rolled backward, as a bouncing sound played. For the multimodal task, infants were shown two alternating geometric objects (round or rectangular, each presented for 5 s) sequentially on either side of the computer screen. Each object was paired with a specific sound (i.e., bounce or ding). Each object moved in synchrony with its sound – the bouncing object moved up and down like a bouncing ball, and the ringing object moved left and right like a bell. For the object solidity task, there were two drawbridges in the center of the screen that fell to the left and right simultaneously with a creaking door sound, and either bouncing or squishing a red block or blue ball (accompanied by the corresponding bouncing or squishing sound).

Each task had a familiarization version and a switch test trial version, which differed in one feature compared to the familiarization version: switched object color (previously yellow objects now became blue, and vice versa; *probability sampling*), switched motion (previously stuck object now bounced off the median, and vice versa; *motion trajectory*), switched sound (previously bouncing object with accompanying sound now rang and moved like a bell, and vice versa; *multimodal binding*), and switched object property (previously squished object now made the drawbridge bounce, and vice versa; *object solidity*). The bouncing and squishing action started at 5 s into the movie. Half of the infants saw one version during familiarization and the other version as a switch test trial, whereas the other half of the infants saw the opposite. Three of the events (probability sampling, motion trajectory, and object solidity) presented simultaneous actions in each of the videos (e.g., a yellow box and a blue ball simultaneously fell in the probability sampling video), while one of the events (multimodal binding) alternated between two actions (e.g., the round object would ding, followed by the rectangular object bouncing). All stimuli were made by the first author with Adobe Flash, and the videos are available on Databrary. Each movie in every task was displayed for 10 s (similar to Wu and Kirkham, 2010). All events were counterbalanced for all infants (i.e., Event A for one infant was Event D for another infant).

For the probability sampling task, infants by 8 months of age likely detect color changes in this deterministic task, as they can distinguish colors in a more complex probabilistic task (Xu and Garcia, 2008). For the motion trajectory task, infants as young as 4 months detect unexpected motion trajectories (Kim and Spelke, 1992; Spelke et al., 1995). For the multimodal binding task, infants as young as 3 months of age detect changes in object-sound-motion mappings (e.g., Kirkham et al., 2012). For the object solidity task, infants as young as 3–4 months of age have been shown to detect events that violate their expectations in this task (e.g., Baillargeon, 1987).

### Design and Procedure

The video stimuli were displayed full-screen on one 17-in monitor, and infants’ fixations were recorded using a Tobii-1750 eye-tracker^1^ integrated with the monitor. Sounds were played via external speakers. The experimental session consisted of four blocks. The first three blocks, which distinguished the two experimental groups (Mixture or Uniform), displayed three events either in uniform blocks (A^∗^18 trials, B^∗^18, C^∗^18) or in randomized blocks (1 trial each: ABCBCA…) (Figure 1). The final block (D^∗^18 trials) was a uniform presentation of a novel event (D) for both groups. In the Mixture condition, the same event was never presented twice in a row. To measure specific learning, switch test trials (hereafter, test trials) were presented after every 9th familiarization trial (i.e., twice per block). In the Mixture condition, the test trial was never related to the familiarization trial that immediately preceded the test trial (e.g., if Event A was presented as the 9th familiarization trial, the switch trial presented next was relevant to either Event B or C). This design decision was made to control for learning from only the immediately preceding trial in the Mixture condition. An attention getter (i.e., a video of a laughing baby) was displayed before the first trial of the experiment, and after every 10 trials (i.e., after every test trial) until infants oriented to the screen for 1 s, as monitored automatically in a MATLAB program.

Prior to the start of the experiment, infants’ gaze was calibrated to five screen locations with the standard calibration procedure using the infant Tobii system (e.g., Wu and Kirkham, 2010). All infants were calibrated to at least four locations. Infant fixations, which were recorded at 50 Hz, were summed across each trial in each block for the final analysis (Kidd et al., 2012). Trials with no looking time data (less than 100 ms of looking time data) were removed from the final analysis (1.68% of trials). Although other looking-time studies have used a 2–3 s cutoff for inclusion of trials, this cutoff is relatively arbitrary, dependent on error from hand-coding looking times, and dependent on the stimuli included in the study. We opted to include a 100 ms cutoff for including a trial because 100 ms is the standard minimum cutoff for fixations, and trials with at least one fixation were included in our analyses. The entire experiment lasted 17 min while the infants sat on the caregiver’s lap 50 cm from the eyetracker.

## Results

### Measures and Analysis Plan

The measure of abstract learning was dishabituation to Event D (novel uniform presentation structure for the Mixture condition, but familiar uniform structure for the Uniform condition) on trials 1–9 and 11–19 in the final test block. Specific learning was measured via dishabituation to the switch test trials that occurred on trials 10 and 20 within each block. A linear mixed-effects regression model was used to analyze the effects of condition (Mixture vs. Uniform, sum-coded), within block trial (1st to 10th trial in every half block, centered), test trial (expected vs. actual looking time on the 10th trial, sum coded), block type (Blocks 1–3 vs. Block 4, sum coded), and block number (looking time slope from Blocks 1–4, centered) on infant looking time. A moving average of looking time across three trials was calculated for trials 1–9 (familiarization trials) for every half block (9 familiarization trials + 1 test trial). Looking time to switch test trials was not included in the moving average. Therefore, the dependent measure consisted of both the moving average values from the familiarization trials, and separately, the actual looking time during the test trials. All of these variables, including two- and three-way interaction terms between Condition and other variables (i.e., Test trial, Block Type, Block Number, and Within block trial), were entered as fixed effects to capture population-level effects. Participant-level random intercepts and random slopes for within block trial and block number also were entered into the regression model to account for individual-level variations. In our analysis, the fixed effects are of theoretical interest, as they reveal the average learning behavior of infants. The random effects serve as a mechanism to control for variability in the data that can be attributed to arbitrary individual differences, thereby improving the reliability of the fixed effects.

The mixed-effects regression model was fitted using the lmerTest package available in R (Kuznetsova et al., 2016). We followed the practice of starting with the maximal random-effects model that is justified by the design and that can converge in a realistic manner (Barr et al., 2013). Then, through a series of pairwise likelihood ratio tests that determine the necessity of including various random effects, the resultant model was overall the best model to describe the data with a minimum number of parameters.

### Explanation and Justification of Mixed-Effects Regression Analyses

Although collapsing the data into means and conducting ANOVAs and standard pairwise *t*-tests may be the historical standard for looking time studies, the benefits of mixed-effects regression models over ANOVAs are documented elsewhere (e.g., Krueger and Tian, 2004; Wainwright et al., 2007). For this study in particular, there are at least three reasons that justify a mixed-effects regression. First, mixed-effects regression addresses the issue of repeated measures through the use of subject-level random effects, which controls for the idiosyncratic differences between subjects and thus further improves the accuracy and reliability of the fixed effects. Second, the mixed-effects regression model allowed us to simplify the analyses into one model with one dependent variable (looking time). Unlike an ANOVA, a mixed-effects regression model assesses the difference between the experimental groups without performing *post hoc* tests and quantifies the difference between groups. Using repeated ANOVA tests on the same dataset inflates the Type I error of the overall analysis. Third, time (trial progression) could be treated as a continuous variable in our analyses, rather than as a discrete/categorical variable, such as familiarization trial versus test trial, which allowed us to better examine the dynamics of learning behavior.

### Main Findings

**FIGURE 3:**
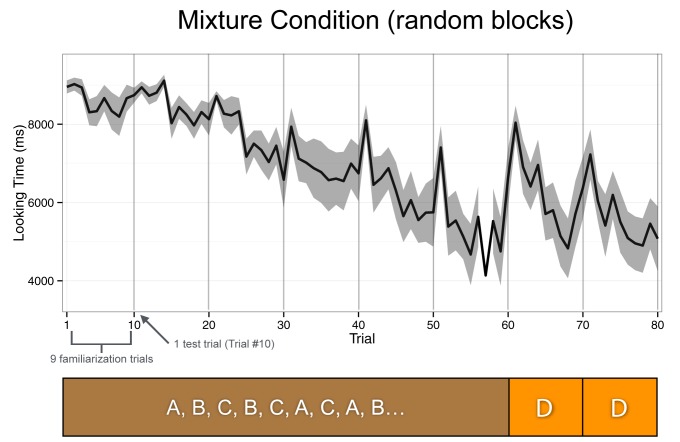
Raw looking times across trials throughout the Mixture condition. The test trials are depicted by the vertical lines (trials 10, 20, 30, etc.), and the familiarization trials were trials 1–9 in every half block.

**FIGURE 4:**
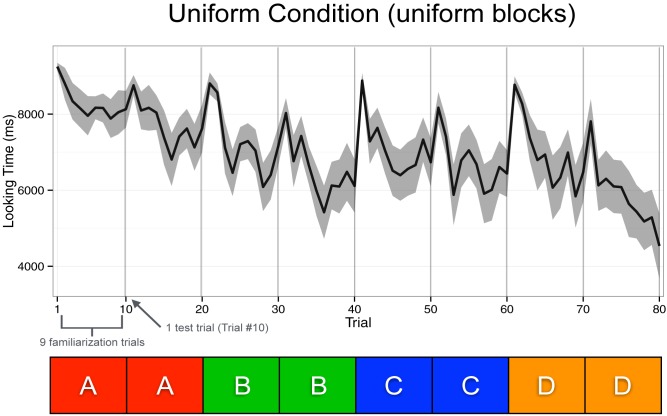
Raw looking times across trials throughout the Uniform condition. The test trials are depicted by the vertical lines (trials 10, 20, 30, etc.), and the familiarization trials were trials 1–9 in every half block.

There were two main findings related to specific and abstract learning, and two supplementary findings, summarized in Table 1. Figure 3 and 4 display the raw looking times across trials throughout the Mixture and Uniform conditions, respectively. At the end of the 10th trial (end of a half block) and before the first trial in the next half block, we played a laughing baby video to recapture infants’ attention, which is represented by the dramatic increase in looking time at the beginning of every half block.

**Table 1:** Fixed effects of the regression model.

	Effect size (ms)	Std Error	*t*-value	*p*-value
(Intercept)	7422.97	237.95	31.196	<0.001^∗^
Condition	186.07	475.89	0.391	0.697
Test Trial	588.33	166.85	3.526	<0.001^∗^
Block Type	696.74	179.33	3.885	<0.001^∗^
Block Number	–487.57	53.04	–9.192	<0.001^∗^
Within Block Trial	–197.49	20.80	–9.494	<0.001^∗^
Test trial × Block Type	–394.17	294.81	–1.337	0.181
Condition × Test Trial	312.60	333.70	0.937	0.349
Condition × Block Type	1102.56	358.65	3.074	0.002^∗^
Condition × Block Number	–330.27	106.08	–3.113	0.003^∗^
Condition × Within Block Trial	29.18	41.60	0.701	0.487
Condition × Test Trial × Block Type	756.86	589.62	1.284	0.199


*^∗^p < 0.01.*

#### Specific Learning in Both Mixture and Uniform Conditions

The first main finding was that there was no significant effect in looking time on the test trials in the last block (trials 70 and 80) between the Mixture and the Uniform conditions (see Condition × Test Trial × Block Type in Table 1, *p* = 0.20). In fact, infants in both conditions looked longer than expected on *all* of the switch test trials (10th trial of every half-block) by an average of 588.33 ms per trial (Test Trial, *p* < 0.001), without any difference between conditions (Condition × Test Trial, *p* = 0.35) or Condition and Block Type (Condition × Test Trial × Block Type, *p* = 0.20). This result suggests that infants in both conditions may have dishabituated similarly during test trials throughout the entire experiment (i.e., specific learning), despite differences in the presentation structure of the first three blocks between the Mixture and Uniform conditions, and between the first three blocks and last test block in the Mixture condition.

#### Abstract Learning in Both Mixture and Uniform Conditions

The second main finding was that infants across both conditions looked an average of 697 ms longer per trial across all familiarization and test trials during the last block compared to the first 60 trials (see Block Type in Table 1, *p* < 0.001). However, this average number is potentially misleading, as this effect is completely driven by infants in the Mixture condition, who looked an average of 1248 ms longer during the last block (*p* < 0.001). By contrast, infants in the Uniform condition looked at the final D blocks with more or less the same interest (145 ms longer, *p* > 0.5). This pattern is what was expected if infants in both conditions showed abstract learning. Abstract learning in the Mixture condition should lead infants to discriminate the mixed ABC structure of the first three blocks from the D-only uniform structure of the final block (i.e., show substantial recovery of looking time to that final block). Abstract learning in the Uniform condition should lead infants to expect that the first three A-only, B-only, and C-only blocks would be followed by a D-only block (i.e., minimal recovery of looking time to that final block). This differential pattern in the behavioral outcome to the two conditions can be seen in a significant interaction effect listed in Table 1 (Condition × Block Type), where infants in the Mixture condition looked 1102 ms longer per trial during the last block than infants in the Uniform condition (*p* = 0.002).

#### Supplementary Findings

The first supplementary finding is that infants in both conditions looked less across trials 1–9 and 11–19 (i.e., within a half block, Within Block Trial), without an effect of Condition (i.e., no significant effect for the Condition × Within Block Trial interaction). This finding demonstrates the typical habituation outcome with a steady decrease in looking time across repeated trials regardless of presentation structure within each half-block of 10 trials (i.e., no significant effect between a repeated single event or three repeated events presented in random order). The second supplementary finding is that all infants looked less as blocks progressed from the first to fourth (Block Number, *p* < 0.001). There was also an effect of Condition here (Condition × Block Number), where infants in the Mixture condition lost interest faster (i.e., displayed a more negative looking-time slope) compared to infants in the Uniform condition (*p* = 0.003).

## Discussion

The present study examined whether novel abstract learning (i.e., presentation structure of audio-visual events) disrupts novel specific learning (i.e., detecting changes in the audio-visual events). During the familiarization phase, 8- to 11-month-old infants were presented with three different audio-visual events, followed by a fourth event in the abstract learning test phase. Infants in the Mixture condition were shown the trials from the first three events in a random order (e.g., ABCBCACAB…), whereas infants in the Uniform condition were shown the trials in a uniform order (first all of the trials from Event A, then the trials from Event B, then Event C). The trials in the fourth event (Event D) were uniform for infants in both conditions. Therefore, infants in the Uniform condition viewed no change in the presentation structure across all four blocks, whereas infants in the Mixture condition switched from a random stimulus order in the first three blocks to a uniform order in the final block. Recovery of looking time during the final block when infants in both conditions saw a uniformly presented novel event was used as the measure of abstract learning. Infants in both conditions exhibited clear evidence of abstract learning: in the Mixture condition by recovering looking time to the D-only test block after familiarization to ABC blocks, and in the Uniform condition by not showing a recovery of looking time to the D-only test block after familiarization to A-only, B-only, and C-only blocks.

In terms of specific learning, at the end of every nine trials, a switch test trial assessed specific learning by changing one feature in each of the events (e.g., color, motion trajectory). We found evidence of specific learning throughout the experiment, including in the final test block when a new event (Event D) was introduced. We also did not find evidence that abstract learning (i.e., increased overall looking due to a switch in presentation structure for the Mixture condition) disrupted later specific learning: There was no significant change in looking time during the last two switch trials compared to earlier switch trials in the Mixture condition and compared to the last two switch trials in the Uniform condition. Our results suggest that when presented with both specific and abstract information to learn, infants learn both types of information in parallel, and abstract learning may not interfere with specific learning. The idea that young learners are open-minded about learning a variety of regularities is supported by other research from Sloutsky and colleagues (e.g., Plebanek and Sloutsky, 2017) and Gopnik and colleagues (e.g., Gopnik et al., 2017).

Although our findings support these overall interpretations, we cannot know if they will generalize to all contexts where specific and abstract learning could interact. One limitation in the current study is that given stimulus constraints, our paradigm could not accommodate fully counterbalancing the experimental design. We did not include a condition that provided infants with mixed presentations throughout the entire experiment because it would not have had a meaningful counter condition that included uniform blocks in the first three habituation phases and a mixed phase at the end. The latter condition would have required three additional events not displayed to infants who were shown mixed presentations throughout. It may be the case that infant learning could be disrupted when switching from a uniform to mixed presentation. However, this procedure may “scaffold” infant learning by presenting an easier learning situation prior to a more difficult learning situation. In our study design, any indication of abstract learning in this hypothetical condition would have been confounded by the novel events. We opted for our design because the stimuli in both conditions of our experiment were identical and only differed in the way they were presented during the familiarization phase, allowing us to draw conclusions based on identical test stimuli in the final block. Future research should investigate the constraints of maintaining learning despite changes in presentation structure.

Another limitation of the study is the relatively small sample size of 16 infants per condition and wide age range in our sample. Because this study was among the first of its kind, it was exploratory. On the one hand, some of our findings are aligned with prior research that used similar sample sizes: we found evidence of specific and abstract learning. On the other hand, we did not find evidence that abstract learning disrupts specific learning, which is the first such demonstration and needs to be replicated. As a starting point for future investigations, our findings may encourage future work to determine the potential costs of abstract knowledge in different domains (e.g., language learning; Gerken, 2006, 2010). Future studies can investigate age effects, because younger vs. older age groups may or may not differ in whether and how abstract learning may disrupt specific learning. In addition, our study allowed infants to acquire expectations about presentation structure prior to the change in structure. However, these expectations were not rooted in real-world experience prior to the experimental session, and therefore may have been dismissed more easily during the experimental session. Future research should investigate whether our effects generalize to situations involving more real-world experience, such as face processing (specific faces vs. race) and language acquisition (specific language content vs. speaker preference).

The infants in our study learned the specific events in both the Uniform and Mixture conditions. In general, repetition of events likely enhances learning during infancy, one of our original hypotheses. Indeed, the presentation structure of many infant habituation studies would support this notion. It may be the case that the events in our paradigm were simple enough to learn even in a mixed sequence, especially given that we only mixed three events in the Mixture condition. In addition, the events were repeated throughout the Mixture condition, even though the repetition structure was not predictable. Task difficulty, specific learning, and presentation structure likely interact in interesting ways. Perhaps our study included relatively easy tasks, whereas including more difficult tasks may lead to no specific learning, even in repeated presentations. In real-world situations that may be more difficult to grasp, perhaps immediate repetition may facilitate learning during infancy more than mixed events. However, the natural learning environment does include situations that resemble the mixture condition. Therefore, perhaps infants within the first year of life adapt to learning in both repeated and mixed conditions. Future research could expand the types of stimuli included and vary task difficulty to investigate this issue.

The costs and benefits of the interaction between abstract and specific learning have potential downstream effects for the efficiency and flexibility of future learning. On the one hand, in cases where abstractions (e.g., rules and contexts) are familiar and infants have to learn about specific novel events (e.g., object features), it would be advantageous for infants to use familiar abstractions to infer the meaning of a novel event. Indeed, prior research has shown that infants learn about specific novel events by using familiar higher-level abstract information (e.g., repetition of visual arrays, Tummeltshammer and Amso, 2018; speech vs. tones, Marcus et al., 2007). On the other hand, when the abstract information is unfamiliar, infants should remain flexible by learning both abstractions and specific events in parallel, rather than applying an inappropriate familiar abstraction to a specific instance. In other words, while abstract knowledge can be a powerful tool to constrain learning (i.e., helping infants determine what to learn), applying erroneous assumptions and constraints could lead the learner to make incorrect inferences about which events to encode, and perhaps what aspects of an event to encode. The finding from the present study suggests that as infants learn abstract information (i.e., generalizing across specific instances), they also may continue acquiring information from specific instances with relatively few assumptions (i.e., without applying much abstract knowledge acquired from prior experiences). Learning both specific and abstract information in parallel may be one reason why infants are proficient in some tasks that are difficult for older populations (Wu et al., 2017).

## Data Availability

The datasets generated for this study are available on request to the corresponding author.

## Ethics Statement

This study was carried out in accordance with the recommendations of the University of Rochester Research Subjects Review Board with written informed consent from all parents of infant subjects. All parents of infant subjects gave written informed consent in accordance with the Declaration of Helsinki. The protocol was approved by the University of Rochester Research Subjects Review Board.

## Author Contributions

RW and RNA came up with the idea. RW created the stimuli and tested the infants. TQ and RW analyzed the data. RW wrote the first draft of the manuscript. All authors edited the manuscript.

## Conflict of Interest Statement

The authors declare that the research was conducted in the absence of any commercial or financial relationships that could be construed as a potential conflict of interest.

## Footnotes

1 www.tobii.com
